# Non-lethal fungal infection could reduce aggression towards strangers in ants

**DOI:** 10.1038/s42003-023-04541-7

**Published:** 2023-02-16

**Authors:** Enikő Csata, Luca Pietro Casacci, Joachim Ruther, Abel Bernadou, Jürgen Heinze, Bálint Markó

**Affiliations:** 1 Institute for Zoology, University of Regensburg, Universitätsstraße 31, D‐93040 Regensburg, Germany; 2 Hungarian Department of Biology and Ecology, Babeș-Bolyai University, Clinicilor 5-7, 400006 Cluj-Napoca, Romania; 3 Department of Life Sciences and Systems Biology, University of Turin, via Accademia Albertina 13, 10123 Torino, Italy; 4 Centre de Recherches sur la Cognition Animale (CRCA), Centre de Biologie Intégrative (CBI), Université de Toulouse, CNRS, UPS, 31062 Toulouse, France; 5 3B Centre for Systems Biology, Biodiversity and Bioresources, Faculty of Biology and Geology, Babeș-Bolyai University, Clinicilor 5-7, 400006 Cluj-Napoca, Romania

**Keywords:** Behavioural ecology, Chemical ecology, Animal behaviour

## Abstract

Many parasites interfere with the behaviour of their hosts. In social animals, such as ants, parasitic interference can cause changes on the level of the individual and also on the level of the society. The ant-parasitic fungus *Rickia wasmannii* influences the behaviour of *Myrmica* ants by expanding the host’s nestmate recognition template, thereby increasing the chance of the colony accepting infected non-nestmates. Infected ants consistently show an increase of the alkane tricosane (*n*-C23) in their cuticular hydrocarbon profiles. Although experimental application of single compounds often elicits aggression towards manipulated ants, we hypothesized that the increase of *n-*C23 might underlie the facilitated acceptance of infected non-nestmates. To test this, we mimicked fungal infection in *M. scabrinodis* by applying synthetic *n*-C23 to fresh ant corpses and observed the reaction of infected and uninfected workers to control and manipulated corpses. Infected ants appeared to be more peaceful towards infected but not uninfected non-nestmates. Adding *n*-C23 to uninfected corpses resulted in reduced aggression in uninfected ants. This supports the hypothesis that *n*-C23 acts as a ‘pacifying’ signal. Our study indicates that parasitic interference with the nestmate discrimination of host ants might eventually change colony structure by increasing genetic heterogeneity in infected colonies.

## Abstract (web summary)

The application of alkane tricosane (*n*-C23) to non-nestmates decreases aggression towards them in ants, in line with its high level in infected workers, pointing at a pacifying signal in parasitic interferences in ant nestmate discrimination.

## Introduction

Parasites often affect the behaviour, morphology and/or physiology of their hosts in various degrees^1–6^. Parasite-induced modifications are generally quite well-defined in the case of individuals^2,7,8^. In social organisms, though, substantial changes can also occur on the level of the society but are less obvious unless the social context is considered^9^. For example, such modifications might impair the capability of social animals to discriminate between related and unrelated individuals^10^. Sociality is typically founded on this capability to prevent non-relatives from benefiting from within-group altruistic actions without reciprocating^11^.

In social insects, the main recognition cues and signals are chemical substances^12–14^, although other signalling modes may also be of importance (vibroacoustic e.g.,^12,15,16^; visual^17,18^; tactile^19^). The discrimination between strangers (both con- and allospecific) and nestmates is largely based on complex mixtures of long-chain cuticular hydrocarbons (CHC), which cover the surface of individuals. Cuticular profiles can consist of more than 100 different hydrocarbons, mostly linear alkanes, methyl-branched alkanes, and alkenes^20^. Both qualitative and quantitative differences in these profiles are used by social insects to recognize outsiders^21–24^. According to the ‘Gestalt’ model, the chemical profiles of the nestmates contribute to the formation of a colony-specific odour^25^ and recognition signals are usually exchanged and homogenised among colony members via passive contact, allogrooming, or trophallaxis, but each individual retains its own chemical profile. Besides colony identity, this profile may encode additional information such as age, task, and sex^26–31^. It serves as a template during the recognition process and helps social insects to prevent potential freeloaders and parasites from entering their colonies^32^.

Many parasites evolved mechanisms to infiltrate insect societies, including passive or active mimicry of host CHC profiles^33,34^. For example, larvae of *Maculinea* butterflies are known to synthesize CHCs specific to their host ants^35,36^, while queens of the slave-making genus *Polyergus* almost instantly acquire CHCs through physical contact with their hosts^37^. Several fungi, viruses, mites, or tapeworms may elicit changes in the CHC profiles of their hosts within a few hours after infection^38–42^. Usually, these changes are restricted to a few infected individuals.

In contrast, the ectoparasitic, non-lethal laboulbenian fungus *Rickia wasmannii* can infect the entire colony without killing it^43,44^ but changing the phenotype of infected individuals^43,45–48^. Besides behavioural and morphological alterations, *R. wasmannii* interferes with the CHC profile of its *Myrmica scabrinodis* host ants^46^. It increases the variation of CHC profiles among infected colony members and opens the infected society towards strangers, such as infected non-nestmates, foreign queens, and parasitic *Maculinea* larvae. This could cause substantial modifications on the social level, as revealed by our previous study^46^. In addition to the higher variability in their CHC profiles, compared to uninfected workers, infected individuals show a consistent, significant increase in the relative abundance of two linear alkanes, *n*-C23 and *n*-C24, with the most pronounced changes in *n*-C23. While *n*-C24 represents only 1–2% of the overall proportion of cuticular hydrocarbons, *n*-C23 contributes about 14% in uninfected workers and 19% in infected ones, suggesting its potential importance in shaping behaviour^46^.

**Fig. 1:**
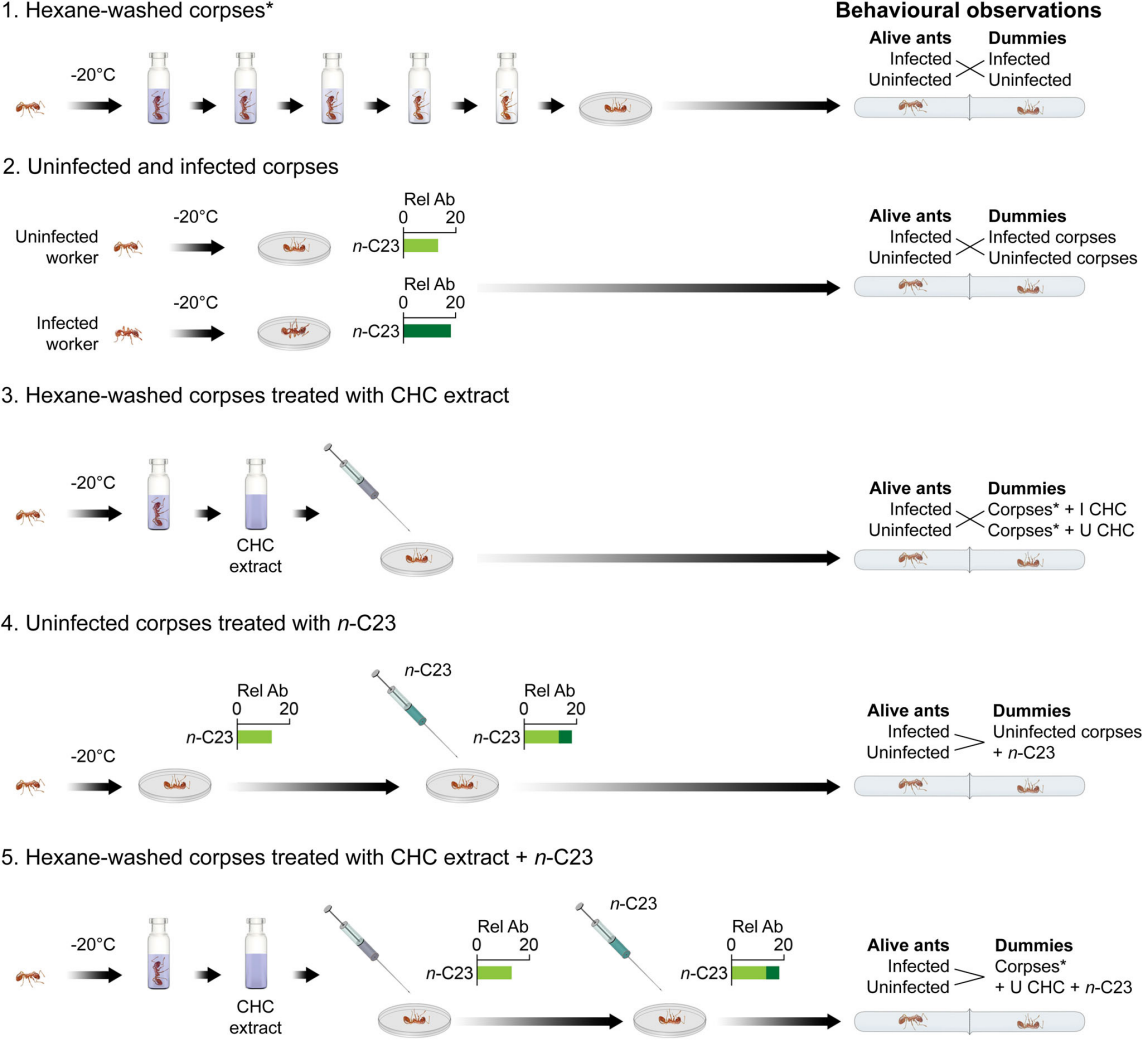
Schematic representation of the overall experimental design. The graph shows the five different behavioural experiments which were performed during our study. (1) Corpses were washed five times and the response of ants to hexane-washed dummies was observed. (2) The reactions of live workers were observed towards infected and uninfected dummies. (3) Corpses were washed (see 1 herein), and cuticular extracts from uninfected or infected workers were applied to them. The behavioural responses of live workers were observed towards these dummies, which were obtained from uninfected colonies. (4) The reaction of workers towards uninfected dummies treated with synthetic *n*-C23. (5) The reactions of workers to hexane-washed dummies onto which we had applied cuticular extracts from uninfected workers supplemented with synthetic *n*-C23. I CHC = cuticular hydrocarbon extracts from infected individuals. U CHC = cuticular hydrocarbon extracts from uninfected individuals. Hexane washed corpses (uninfected) are marked with an asterisk (*). Rel Ab = Relative Abundance. The majority of the elements (vial, Petri-dish, syringe) were created by the authors, using freely available elements on the internet (Petri-dish, syringe, vial). The *Myrmica* ant icon was created by Ms. Natalia Timuș, our previous co-author, who gave her consent to the free use of the icon within the manuscript.

Previous studies in social Hymenoptera have shown that adding single components to the CHC profile elicits higher aggression^49–52^. In contrast, *Myrmica* ants reacted less aggressively to ants infected with *R. wasmannii*, despite the increased percentage of *n-*C23, making it a strong candidate for a pacifying compound. Here, we examine by experimental manipulation of the CHC profiles whether (a) the surplus *n*-C23 linked to infection makes the infected host less aggressive towards others, or (b) it acts on the opponent by pacifying it irrespective of the opponent’s health status (Fig. 1).

## Results

The results of the barcoding analyses allowed us to state that the study population undoubtedly belonged to *M. scabrinodis* and fell into a single lineage indicated as *M. scabrinodis* type A^53,54^ (see DNA barcoding in Supplementary Results; Supplementary Fig. 2).

### Baseline aggression

Almost no aggressive interactions occurred in the control experiments (GLM, *p* = 0.99, *N* = 66, Supplementary Table 2, Supplementary Figs. 3 and 4). Neither uninfected, nor infected *M. scabrinodis* workers showed any aggression towards dummies with removed CHC (*N* = 36). This confirmed the role of CHC profiles in the discrimination process and demonstrated the suitability of the applied CHC-removal procedure in producing ‘odourless’ dummies for further experimental manipulations.

**Fig. 2:**
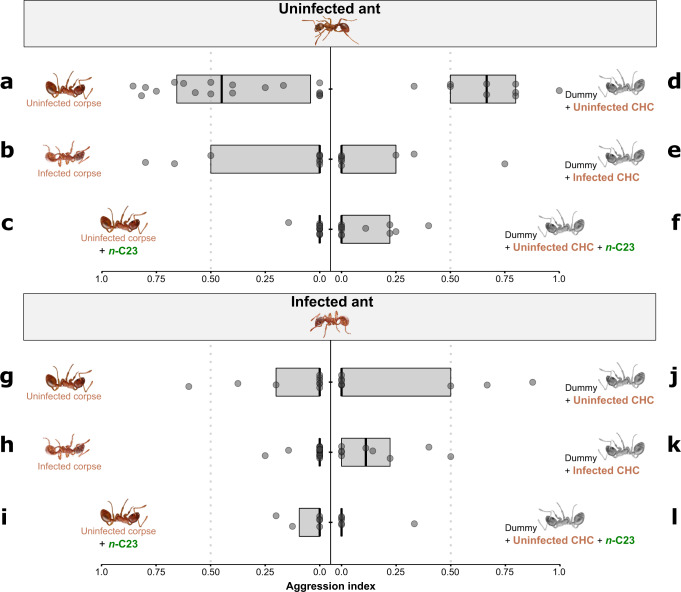
Aggression indices resulting from interaction assays between live uninfected and infected *M. scabrinodis* workers and different types of dummies. The upper part of the graph shows the reactions of uninfected ants towards (**a**) Uninfected corpse; (**b**) Infected corpse; (**c**) Uninfected corpse + *n*-C23; (**d**) Dummy + uninfected CHC; (**e**) Dummy + infected CHC; (**f**) Dummy + Uninfected CHC + *n*-C23. The lower part of the graph shows the reactions of infected ants towards (**g**) Uninfected corpse; (**h**) Infected corpse; (**i**) Uninfected corpse + *n*-C23; (**j**) Dummy + uninfected CHC; (**k**) Dummy + infected CHC; (**l**) Dummy + Uninfected CHC + *n*-C23. Dummies are coloured in grey, while corpses are coloured in dark brown. Box-and-whisker plots with median, 25–75% quartiles, maximum/minimum range and outliers. The *Myrmica* ant icon was created by Ms. Natalia Timuș.

The application of CHC extracts originating from either infected or uninfected workers to CHC-removed dummies resulted in responses by live individuals comparable to corresponding unmanipulated assays in almost all cases, confirming the accuracy of the CHC-transfer method (Fig. 2; Supplementary Table 4): U–I corpse vs U–I extract GLM p = 0.54 (Fig. 2b, e); U–U corpse vs U–U extract GLM *p* = 0.36 (Fig. 2a, d); I–U corpse vs I–U extract GLMM *z* = −0.85, *p* = 0.39 (Fig. 2g, j). In a single case we detected significant difference (I–I corpse vs I–I extract, GLM *p* = 0.03; Fig. 2h, k) showing that the application of extracts might not always produce identical effects to natural situation. However, the comparison of U-U extract vs I-I extract (see Supplementary Table 4; Fig. 2d, k) did result in significant effects in line with unmanipulated observations (see below).

In unmanipulated assays, the level of aggression was significantly lower when both ants were infected compared to uninfected counterparts (I–I corpse vs U–U corpse GLM, *p* = 0.0001; Fig. 2a, h; Supplementary Table 4). Generally, infected ants were less aggressive towards both infected (GLM, *p* = 0.002; Fig. 2b, h; Supplementary Table 4), and uninfected corpses (GLM, *p* = 0.05; Fig. 2a, g; Supplementary Table 4) than healthy ones. Infected ants treated infected corpses less aggressively compared to uninfected ones (GLM, *p* = 0.01; Fig. 2g, h; Supplementary Table 4), while healthy workers did not differentiate between cadavers of different health status (GLM, *p* = 0.15; Fig. 2a, b; Supplementary Table 4).

### Manipulation of the CHC profiles

The aggression of infected workers was significantly lower towards dummies supplemented with cuticular extract from infected individuals in comparison to assays where both were uninfected (GLM, U-U extract vs I-I extract, *p* = 0.01; Fig. 2d, k; Supplementary Table 4), confirming the pattern observed in unmanipulated situation.

Manipulation of CHC profiles in any further combinations resulted in significant changes in the reaction of live ants, especially of uninfected ones, yielding very similar results.

Thus, contrary to what was observed in unmanipulated assays, uninfected ants responded with a significantly reduced aggression towards hexane-washed dummies treated with cuticular extract from infected individuals than to uninfected dummies (GLM, U–I extract vs U–U extract, *p* = 0.008, Fig. 2d, e; Supplementary Table 4). No significant differences were detected in any other comparisons (I–I extract vs I–U extract; I–U extract vs U–U extract; I–I extract vs U–I extract; see Supplementary Table 5). Also the addition of *n*-C23 to uninfected dummies decreased significantly the level of aggression displayed by healthy ants (GLM, U–U + *n*-C23 vs U–U, *p* = 0.001; Fig. 2a, c Supplementary Table 4). In contrast, no differences were observed between the assays when the live counterpart was already infected (GLMM, I-U vs I–U + *n*-C23 z = 1.49, *p* = 0.13; Fig. 2g, i; Supplementary Table 4).

Similar results were obtained when CHC-stripped dummies which were treated with CHC extracts from untreated ants and additional *n*-C23 were used (see Fig. 2). The supplementation with *n*-C23 of hexane-washed dummies treated with CHC extract from healthy ants also changed the outcome of interactions both in uninfected and infected ants by significantly lowering the aggression (GLM, U–U extract vs U–U extract + *n*-C23, *p* = 0.001, Col ID *p* = 0.79, *N* = 21, Fig. 2d, f; I–U extract vs I–U extract + *n*-C23 GLM, *p* = 0.01, Col ID *p* = 0.02, *N* = 15, Fig. 2j, l), underlining again the importance of *n*-C23.

### Chemical analyses of treatment efficiency

In total, 37 hydrocarbons were identified in *M. scabrinodis* reported in Csata et al.^46^ (see Supplementary Table 3). Control extracts from dummies that had been washed five times with hexane contained only traces of four to five CHCs out of 37 being present in non-washed workers (Supplementary Fig. 1). These residues were from those compounds with the highest abundance in non-washed ants^46^. The abundance of these major compounds, however, was 10-15 times lower than in the unwashed ants. Therefore, we concluded that the applied method was suitable for obtaining dummies without relevant amounts of cuticular compounds.

The relative abundance of *n*-C23 in uninfected ants treated with the synthetic alkane was on average 22.4 ± 6.0% (mean ± SD) and did not differ from the relative abundance of *n*-C23 in untreated infected ants (LM *t* = 1.87, *p* = 0.11, *N* = 21), thus confirming that the topical application of *n*-C23 on uninfected ant corpses was successful to achieve the relative abundance of this linear alkane present on infected ants. In untreated, uninfected ants, the relative abundance of *n*-C23 was only 13.8 ± 1.3% and differed significantly from the relative abundance present on uninfected *n*-C23-treated ants (LM *t* = 4.46, *p* < 0.001, *N* = 23).

## Discussion

The current study reveals in social insects the ‘pacifying’ role of a simple component of the cuticular hydrocarbon profile linked to a fungal pathogen. Appeasement effect of certain compounds (e.g., crematoenones) has already been reported in ants^55^, however, it involves two mutualistic ant species that regularly share a common nest. In our case, it is the parasite that induces behavioural changes through modification of the CHC profile.

Our first hypothesis according to which *n*-C23 supplemented (infected) individuals become peaceful toward everyone can be dismissed, since the almost complete lack of aggression occurred towards infected counterparts. However, the surplus of *n*-C23 does seem to elicit less aggression from the interacting partner, thus the second hypothesis is to be confirmed.

There is a variation in the response of ants when it comes to compare reaction to corpses supplemented with *n*-C23 with reaction to dummies supplemented with CHC extracts, however these all show the same tendency even if the magnitudes differ. Methodological limitations as e.g. differences between the applied experimental amounts of CHC extracts vs natural quantity, or minor differences in the age of corpses^56^ might play a role in this. Within-colony genetic heterogeneity might also influence the response of colony members to non-nestmates to a certain degree contributing to the variability observed, even if the overall pattern stays the same.

The increased variation of CHC profile in infected colonies^46^ makes them in general more tolerant towards strangers than uninfected ones as proven by current assays as well. These all can contribute to a higher degree of heterogeneity of infected colonies as already addressed by Csata et al.^46^ which could be advantageous for the fungal parasite. There is a fair chance that *R. wasmannii* tapped into its host nestmate recognition system tampering with a relatively simple security code. There is also a chance that *n*-C23 could have a wider effect in the genus *Myrmica* since *R. wasmannii* is present in many species belonging to this genus. Whether the effect of the compound goes beyond this parasite-host system needs to be addressed in further studies.

In insect societies, recognition of relatives and intruders is crucially important since altruistic actions need to be directed towards relatives. Some hydrocarbon classes seem to be more informative than others for nestmate recognition. In particular, methyl-branched alkanes and linear alkenes seem to provide more information than linear alkanes [e.g., ^49,57,58^], which are thought to be structurally more effective in preventing dehydration^59,60^. The role of alkanes is currently ambiguous and appears to differ between species. In honeybees (*Apis mellifera*), they do not seem to have any effect^61,62^. However, in the ants *Formica japonica*^63^ and *Linepithema humile*^64^, workers were unable to distinguish between non-nestmates and nestmates when only the respective alkane or alkene fraction of the cuticular profile was presented to them. When the chemical profiles of *L. humile* nestmates were supplemented with linear alkanes (from *n*-C23 to *n*-C30 and *n*-C33), the tested workers responded aggressively. A similar behaviour occurred in the workers of the European hornet, *Vespa crabro*^51^.

In the light of the examples given above, the results of the present study are highly remarkable, as they show that *n*-C23 represents a ‘pacification’ cue that, when exceeding normal levels, attenuates aggressive responses in conspecifics. The mentioned changes and effect on the nestmate recognition system may also have an impact on the spread of pathogens through increased tolerance towards non-nestmates and young queens that seek to infiltrate in established colonies^46^. The reduced discrimination accuracy of infected ants might also compromise their ability to detect and remove other social parasites. For instance, a high degree of chemical mimicry has been shown recently in parasitic queens of the workerless inquiline species *Myrmica karavajevi*, which parasitizes *M. scabrinodis* colonies^65^. Interestingly, high percentages of *n*-C23 were detected in the CHC profiles of queens and newly eclosed males of *M. karavajevi* which are almost twice the level found in the CHC profiles of host queens. This high relative abundance of *n*-C23 might thus facilitate the parasite usurpation of host colonies and the safe eclosion of parasite males by reducing the aggressiveness of host workers^65^.

Although the number of investigated colonies is relatively low, the results are straightforward, showing that the pathogen-related relative increase of *n*-C23 could play a crucial role in the nestmate vs non-nestmate discrimination accuracy in *M. scabrinodis*. The reduced aggressiveness caused by *n-*C23 could come along in infected colonies with a hampered ability to discriminate against non-related conspecifics that may facilitate the propagation of *R. wasmannii* and may be exploited by other pathogens and social parasites to sneak into *M. scabrinodis* colonies.

Our work paves the way for future studies investigating whether this phenomenon is conserved across other eusocial insect species and exploring the physiological mechanisms underlying the increased tolerance towards pathogen-infected conspecifics.

## Materials and methods

### Study species and site

Colonies of the ant *Myrmica scabrinodis*, both infected (I, *N* = 6) and uninfected (U, *N* = 6), were collected from the same population near Cluj-Napoca, Romania (46.92 N, 23.73E, 410–460 m a.s.l.) as in the previous study^46^. The collected colonies were kept together with their brood in plastic boxes (16 × 10 × 5 cm) with wet foam bricks under controlled lab conditions in a climate chamber (20 °C, 12 L:12D cycles) with a food mixture of sugar and proteins^66^ provided ad libitum on daily basis.

Infected colonies were parasitized by the ectoparasitic fungus *Rickia wasmannii*, which obligatorily exploits *Myrmica* ants^67–69^. This fungus does not have a mycelium, and its thallus develops from a bicellular ascospore attached to the outer layer of the host cuticle^47,70^. In our study population, the colony-level prevalence of the fungus was more than 50%, while the within-colony prevalence reached 100% in certain colonies^44^. Both infected and uninfected colonies are highly variable with regards to worker number and queen number in the study population^46^.

The species identity of ants and their fungal infection status was assessed in the field and later confirmed in the lab using an Olympus SZ51 stereomicroscope. In the case of the ants, we used the keys of Czekes et al.^71^ for morphological confirmation. As there are two separate lineages within *M. scabrinodis*^53^ that might be good candidates for cryptic species, DNA barcoding was applied to elucidate whether these two lineages exist in the study population. DNA was obtained from adult specimens from infected and uninfected colonies (see DNA barcoding).

Voucher specimen from some studied infected and uninfected colonies along with the list of codes are deposited in the Myrmecological collection of the Babeș-Bolyai University in Cluj-Napoca, Romania.

### General experimental setup

Dyadic behavioural tests^46,72^ were performed after four days of acclimatisation under laboratory conditions with worker ants from infected and uninfected colonies (Fig. 1). The selected individuals were isolated in test tubes for one minute before each test. Individuals showing impaired locomotion or any other behavioural disorder were discarded and replaced with another individual.

Since in dyadic tests with live workers it is hard to tell apart the reactions of the two individuals, we used one live worker and one freshly killed non-nestmate conspecific corpse (dummy) in each assay. This allowed us to precisely characterize the response of the live worker. Fresh dummies, obtained by freeze-killing workers (−20 °C for 1 h), are still perceived by ants as live ones due to the integrity of their CHC profile and the low amounts of linoleic and oleic acids. These two acids are used by the ants to detect dead colony members, but they reach the threshold levels for corpse removal only a few hours after an individual’s death as specifically proven in *Myrmica*^73^. In each test, new workers and dummies were used.

To prove the role of CHC profile in nestmate/non-nestmate discrimination in *M. scabrinodis*, we initially tested (1) the response of ants to hexane-washed dummies (see below for further details; *N* = 36). Then we performed unmanipulated assays (2) in which the reactions of live workers were tested to infected (I) and uninfected (U) dummies (*N* = 54) to assess the baseline aggression towards non-nestmates for both cases (Fig. 1).

In the next step (3), uninfected washed dummies were used for the application of extracts to exclude any tactile and visual cues that might arise from the presence of fungal thalli. We manipulated hexane-washed dummies obtained from workers from uninfected colonies by applying cuticular extracts from uninfected or infected workers (see below for further details) to them, and then we recorded the behavioural responses of live workers (*N* = 90; Fig. 1). Thus, we could reveal whether adding CHC extracts to hexane-washed dummies elicits responses from workers matching responses to untreated dummies of similar infection status.

To elucidate the potential role of *n*-C23 (4), we recorded the reaction of workers towards uninfected dummies treated with synthetic *n*-C23 (*N* = 45; Fig. 1). Finally (5), we also tested the reactions of workers to hexane-washed dummies onto which we had applied cuticular extracts from uninfected workers supplemented with synthetic *n*-C23 (*N* = 45; Fig. 1). As a control, workers from six infected (I) and six uninfected (U) colonies were used to test the aggressiveness at the intracolonial level in each manipulation assay (*N* = 66 tests).

### Behavioural assays

Only old workers (more than 20 days old) were used since they are the most infected individuals in a colony, thus the most affected ones^74^. They also constitute the foraging workforce having an increased chance of meeting rivals and potential intruders and constitute the first line of colony defence. Therefore, their nestmate discrimination ability should be more pronounced than those of younger nurse workers. Old workers were randomly selected based on their location in the nest arena, outside the larval chamber, and their darker cuticular pigmentation (brown), as traditionally used for age class estimation in *Myrmica*^74,75^.

The assays were carried out using two connected transparent plastic tubes (3 cm long), which were initially separated by a small piece of red plastic foil. A single *M. scabrinodis* worker was placed in one tube, while the dummy was placed in the other tube. The worker was allowed to acclimatise for one minute before the red plastic foil was removed. After each assay, the plastic tubes were rinsed with ethanol (98%) and water, then wiped out and left to dry for at least 30 min. Assays were carried out with both infected and uninfected workers with several replicates per combination, and new individuals were used for each test (for the exact test number per combination see Supplementary Tables 1 and 2). The observations started after the first contact of the live individual with the presented dummy and ended after three minutes. All interactions were recorded and categorised as: (1) grooming, (2) antennation, (3) carrying, (4) fleeing/retreating, (5) mandible gaping, (6) biting, (7) dragging, and (8) stinging. Grooming was considered a socio-positive behaviour, antennation and carrying were considered neutral, and the last five behaviours were considered aggressive. We considered dragging when a worker bit on the corpse with its mandibles and pulled or pushed it on the ground. The act could be preceded by aggressive biting and then could be continued in stinging. Unlike dragging, in the case of carrying the worker did not charge the corpse but lifted it above the ground and maintained it in a more or less upright position in a peaceful manner^76^. An aggression index (AI) for each encounter was calculated as follows: AI = the total number of aggressive responses divided by the total number of interactions^46,77^. To eliminate or to reduce observational biases, the observer did not know the infection status of the live worker.

### Preparation of corpses (untreated)

Old uninfected and infected ants were selected randomly from their colonies. After selection, the individuals were freeze killed for 10 min. After thawing the freeze-killed corpses for 20 min at room temperature, they were used for the behavioural assays. The corpses were prepared in identical manner for testing the aggressivity of the infected and uninfected individuals toward nestmate corpses (within colony aggression) and towards non-nestmate corpses (between colony aggression).

### Preparation of hexane-washed dummies

Only uninfected ants from uninfected colonies were used to prepare washed dummies used in behavioural assays. After thawing the freeze-killed corpses for 20 min at room temperature, they were placed in clean glass vials and submerged in 300 μl of hexane and manually shaken. After 5 min the ants were transferred to new clean glass vials and washed again with 300 μl of hexane. This procedure was repeated 5 times. The hexane residues were left to evaporate for 10 min before the dummy was used for the behavioural assays.

### Preparation of hexane-washed dummies supplemented with cuticular extracts

Both infected and uninfected workers were used as dummies. After freeze-killing them, two workers from the same colony were submerged in 200 μl of hexane and manually shaken. The extracts were transferred to clean glass inserts (0.2 ml) after 20 min. The hexane was evaporated under a stream of nitrogen and then the content of each glass insert was resuspended in 15 µl of hexane by rinsing the walls of the insert repeatedly using a glass syringe (25 µl, Hamilton, Bonaduz, Switzerland). The resuspended cuticular extract was then taken with another glass syringe (10 µl, Hamilton, Bonaduz, Switzerland). The tip of the syringe needle was then carefully attached to a hexane-washed dummy, and it was carefully raised in the air with the needle, which allowed the formation of an intact and uniform droplet around the dummy in the air. The hexane was allowed to evaporate for 10 min before the dummy was used for the behavioural assays.

### Preparation of uninfected ant dummies supplemented with *n*-C23

Only uninfected workers were used as dummies. We prepared a hexane solution of *n*-C23 (8 ng/μl, Supelco, Bellefonte, Pennsylvania, Sigma Aldrich), and 10 µl (representing a dose of 80 ng) were then taken with a glass syringe (10 µl, Hamilton, Bonaduz, Switzerland). The tip of the syringe needle was then carefully attached to the uninfected corpse, and it was carefully raised in the air with the needle, which allowed the formation of an intact and uniform droplet around the dummy in the air. The hexane was allowed to evaporate for 10 min before the behavioural assays. The dose of *n*-C23 applied to uninfected dummies (80 ng) was adjusted to shift its relative abundance to the levels found on the cuticle of ants infected by *R. wasmannii* (18.72 ± 5.25%;^46^).

### Hexane-washed corpses treated with cuticular extracts from uninfected ants and *n*-C23

Hexane-washed uninfected corpses were treated with cuticular extracts from uninfected ants as described previously. After drying, they were treated additionally with 10 µl (representing 80 ng) of the *n*-C23 solution. Ants were carefully raised in the air with the needle, which allowed the formation of an intact and uniform droplet around the corpse in the air. The hexane was allowed to evaporate for 10 min under a stream of nitrogen before the behavioural assays.

### Posterior verifications of CHC profiles

Previously 37 hydrocarbons were identified in *M. scabrinodis*^46^ (see Supplementary Table 3). Cuticular extracts from ant corpses that had been washed five times with hexane (dummies) were analysed to verify that the treatment had successfully removed most of the CHC profile. We also analysed the relative abundance of *n*-C23 in the CHC profile of uninfected workers treated with *n*-C23 to check whether it was similar to the levels found in infected ants (see Supplementary Fig. 1). We individually extracted the CHC profile of dummies (*N* = 4) and treated ants (*N* = 6) for 20 min in 200 μl of hexane. Subsequently, the extract was transferred to a clean glass insert (0.2 ml) and 8 µl of a hexane solution of *n*-eicosane (*n*-C20, Supelco, Bellefonte, Pennsylvania) (100 ng/µl, representing 800 ng) was added to each extract as an internal standard. Samples were then carefully dried under a stream of nitrogen before being re-suspended in a final volume of 20 μl of hexane. Two μl of each sample were analysed on a Shimadzu GC2010 gas chromatograph (GC) connected to a QP2010 plus mass spectrometer (MS; Shimadzu, Duisburg, Germany). The GC was equipped with a non-polar capillary column (BPX-5, 30 m length, 0.25 mm inner diameter, 0.25 μm film thickness; SGE Analytical Sciences, Milton Keynes, UK). Helium was used as carrier gas with a constant linear velocity of 50 cm s^−1^. The initial oven temperature was 70 °C and ramped at 30 °C/min to 150 °C. It was then increased to 320 °C at a rate of 5 °C/min and held for 10 min at 320 °C. Splitless injection (2 min) was performed at 280 °C. Mass spectra were acquired in full scan mode every 0.3 s with a scan lapse of 0.1 s, the mass was m/z 40 to 600. Mass spectra were acquired in EI mode at 70 eV. The chromatograms were manually integrated to calculate the area of each peak of interest using the proportion of the sum over the area of all peaks.

### DNA barcoding

DNA was obtained from 2 individuals/colony from 3 infected and 3 uninfected *Myrmica scabrinodis* colonies, respectively. The DNA barcoding region was amplified using the LCO1490/HC02198 primer pair based on a standard protocol. The sequences obtained were then analysed together with other sequences of *M. scabrinodis* (lineages A and B; see^53,54^), *M. sabuleti*, *M. vandeli*, and *M. aloba* obtained from Barcode of Life Database (BOLD^78^,) and GeneBank. *Manica rubida* ant species was used as an outgroup. The phylogenetic analysis was carried out in Geneious Prime® 2022.0.1 by aligning the sequences and constructing the neighbor-joining tree with Tamura-Nei distances. The consensus tree was built using the bootstrap resampling method with 1000 replicates.

### Statistics and reproducibility

Aggression indices were analysed at first using a generalised linear mixed model approach (GLMM, binomial error, maximum likelihood fit) with colony identifier (Col ID) of live ants as random factor, and with test combinations (eg. I–I, I–U, etc.) as fixed factors. In the vast majority of the cases, though, singularity issues hindered the application of the model (see Supplementary Tables 4–6). In these cases, we applied generalised linear model approach (GLM, binomial error, maximum likelihood fit) with test combinations and Col ID as fixed factors in order to address the issue of data dependence (see Supplementary Tables 4–6).

Relative abundances of *n*-C23 present on the cuticle of infected and uninfected old workers and experimental ants treated with *n*-C23 were compared using a linear model approach (LM, maximum likelihood fit). Chemical data of infected and uninfected old workers are derived from Csata et al.^46^.

All statistics were performed using R 4.0.2^79^. GLMMs were applied using *glmer* function, while GLMs were performed using *glm* functions in the *lme4* package^80^. For GLMMs and GLMs, test outputs (z and Residual Deviation values, respectively) were obtained using the function *anova* in the *car* package^81^. Tukey’s HSD test was used to calculate post-hoc comparisons on each factor in the model using the function *glht* from the R package *multcomp*^82^. LMs were applied using *lm* function, in the *lme4* package^80^. The graphs were produced using the *ggplot2* R package^83^.

### Reporting summary

Further information on research design is available in the Nature Portfolio Reporting Summary linked to this article.

## Supplementary information

Supplementary Information

Description of Additional Supplementary Files

Supplementary Code 1

Reporting Summary

## Data Availability

All data were uploaded in the Dryad platform (https://datadryad.org/stash/share/DJrVniofT0ftWpYaoWxkUIuVj_WFuG-cm18DwcybPMU).

## Code availability

The R scripts for statistical analyses and for the graphs are featured in the Supplementary files (Supplementary Code 1).
