# Supplementary material for: Non-lethal fungal infection could reduce aggression towards strangers in ants
Source: Commun Biol. 2023 Feb 16;6:183. doi: 10.1038/s42003-023-04541-7 (PMC9935638; doi:10.1038/s42003-023-04541-7)
Supplement: Supplementary file 1 — Supplementary Information [file 42003_2023_4541_MOESM1_ESM.pdf]

Title: Non-lethal fungal infection could reduce aggression towards strangers in ants.

Authors: Enikő Csata*[1,2], Luca Pietro Casacci*[3], Joachim Ruther[1], Abel Bernadou[1,4], Jürgen Heinze[1], Bálint Markó[2,4]

Affiliations

[1]Institute for Zoology, University of Regensburg, Universitätsstraße 31, D-93040 Regensburg, Germany

[2]Hungarian Department of Biology and Ecology, Babeș-Bolyai University, Clinicilor 5-7, 400006 Cluj-Napoca, Romania, *marko.balint@ubbcluj.ro*

[3]Department of Life Sciences and Systems Biology, University of Turin, via Accademia Albertina 13, 10023 Torino, Italy

[4]Centre de Recherches sur la Cognition Animale (CRCA), Centre de Biologie Intégrative (CBI), Université de Toulouse, CNRS, UPS, 31062, Toulouse, France

[5]3B Centre for Systems Biology, Biodiversity and Bioresources, Faculty of Biology and Geology, Babeș-Bolyai University, Clinicilor 5-7, 400006 Cluj-Napoca, Romania

***Corresponding authors**:

Enikő Csata *Eniko.Csata@biologie.uni-regensburg.de*

Luca Pietro Casacci *luca.casacci@unito.it*

These authors contributed equally: Enikő Csata, Luca Pietro Casacci

Keywords: cuticular hydrocarbons, discrimination, fungal infection, manipulation, *Rickia wasmannii*, social cohesion

Experiments

Supplementary Table 1. Sample size per interaction assay in the case of colony aggression (baseline aggression, manipulation of the CHC-profiles). The number of colonies used in each test (reported separately for live individuals and for dummies), the number of tests and the test type are reported.

No. of colonies used for live individuals in each test	No. of live individuals /colony	No. of colonies used for dummies in each test	No. of dummies/ colony	No. of tests performed in each type of experiments	Test type
2	3<br>6	2	3<br>6	9	I-I Extract
3	3<br>3<br>3	3	3<br>3<br>3	9	I-U Extract
3	3<br>3<br>3	3	3<br>3<br>3	9	U-I Extract
2	3<br>6	2	3<br>6	9	U-U Extract
3	6<br>6<br>6	3	6<br>6<br>6	18	I-I Corpse
3	3<br>3<br>3	3	3<br>3<br>3	9	I-U Corpse
3	3<br>3<br>3	3	3<br>3<br>3	9	U-I Corpse
3	6<br>6<br>6	3	6<br>6<br>6	18	U-U Corpse
3	3<br>6<br>3	2	6<br>6	12	U-U Corpse+*n*-C23
3	1<br>2<br>3	2	3<br>3	6	I-U Corpse+*n*-C23
2	3<br>3	2	3<br>3	6	I-U Extract+*n*-C23
3	6<br>3<br>3	2	6<br>6	12	U-U Extract+*n*-C23
3	3<br>3<br>3	3	3<br>3<br>3	9	I-U Washed
3	3<br>3<br>3	3	3<br>3<br>3	9	U-U Washed
3	3	3	3	9	I-I Washed

	3<br>3		3<br>3		
3	3<br>3<br>3	3	3<br>3<br>3	9	U-I Washed

Supplementary Table 2. Sample size per interaction assay in the case of intracolonial aggression (control, within nest). The number of colonies used in each test, the number of tests and the test type are reported.

No. of colonies used for live individuals in each test	No. of live individuals/ colony	No. of colonies used for dummies in each test	No. of dummies/ colony	No. of tests performed in each type of experiments	Test type
3	3<br>3<br>3	3	3<br>3<br>3	9	I-I Untreated
3	3<br>3<br>3	3	3<br>3<br>3	9	I-I Washed
3	3<br>3<br>3	3	3<br>3<br>3	9	I-I Extract
3	3<br>3<br>3	3	3<br>3<br>3	9	U-U Untreated
3	3<br>3<br>3	3	3<br>3<br>3	9	U-U Washed
3	3<br>3<br>3	3	3<br>3<br>3	9	U-U Extract
2	3<br>3	2	3<br>3	6	U-U Corpse+*n*-C23
2	3<br>3	2	3<br>3	6	U-U Extract+*n*-C23

Supplementary Table 3. Cuticular hydrocarbons of *Myrmica scabrinodis* with their relative proportion (±SEM) from Csata et al. [1].

Peak	Hydrocarbon	Relative proportion (±SEM)
1	*n*-C21	0.65 (±0.41)
2	3-MeC21	0.60 (±0.34)
3	*n*-C22	0.71 (±0.27)
4	3-MeC22	0.58 (±0.22)
5	X-C23:1	1.09 (±0.21)
6	*n*-C23	12.71 (±1.81)
7	7-MeC23	0.14 (±0.10)
8	5-MeC23	0.06 (±0.03)
9	3-MeC23	15.21 (±1.21)
10	X-C24:1	0.29 (±0.11)
11	X’-C24:1	0.02 (±0.03)
12	*n*-C24	1.12 (±0.24)
13	8-MeC24	0.51 (±0.24)
14	X,Y-C25:2	0.11 (±0.05)
15	4-MeC24	16.06 (±4.65)
16	X’,Y’-C25:2	35.17 (±3.95)
17	X-C25:1	2.05 (±0.42)
18	X’-C25:1	0.13 (±0.06)
19	*n*-C25	6.44 (±0.82)
20	5-MeC25	0.62 (±0.32)
21	3-MeC25	1.94 (±0.41)
22	5,17 di-MeC25	0.11 (±0.06)
23	*n*-C26	0.12 (±0.05)
24	3,9-diMeC25	0.09 (±0.05)
25	X,Y-C27:2	0.08 (±0.07)
26	X-C27:1	0.10 (±0.04)
27	X’-C27:1	0.85 (±0.20)
28	*n*-C27	0.57 (±0.13)
29	C28 + unknown	0.03 (±0.01)
30	X-C29:1	0.43 (±0.26)
31	*n*-C29	0.49 (±0.19)
32	15-, 13-, 11-MeC29	0.15 (±0.10)
33	5,17-diMeC29	0.14 (±0.11)
34	C30 + unknown	0.02 (±0.01)
35	X-C31:1	0.40 (±0.24)

36	X'-C31:1	0.05 (±0.03)
37	*n*-C31	0.15 (±0.09)

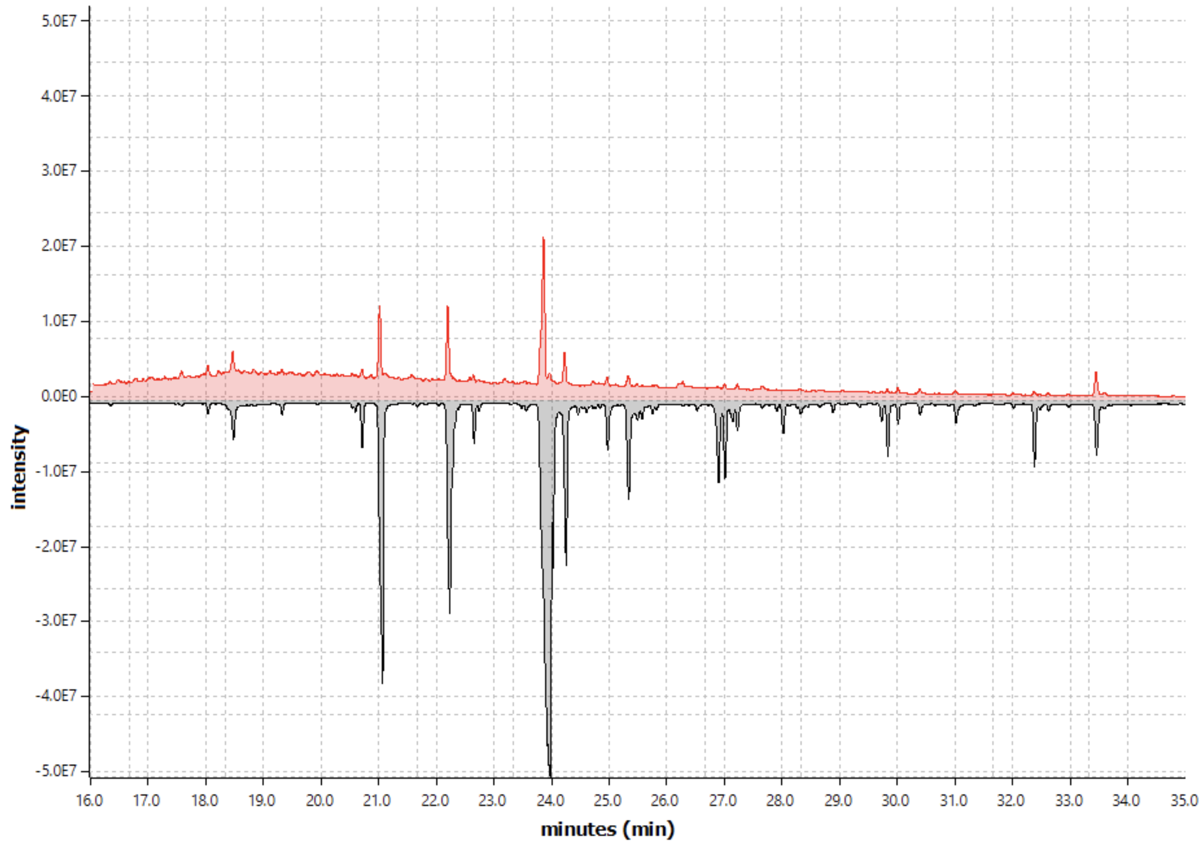

Supplementary Figure 1. The hydrocarbon profiles of an uninfected *Myrmica scabrinodis* worker (in grey) and a dummy (in red). The dummy was an uninfected worker washed 5 times with hexane.

Supplementary results

<u>DNA barcoding</u>

The consensus tree obtained allowed us to state that population included in the present study undoubtedly belonged to *M. scabrinodis* species, and also fell into a single lineage indicated by some authors as *M. scabrinodis* type A [1, 2]. The obtained sequences will be published in a further work in preparation.

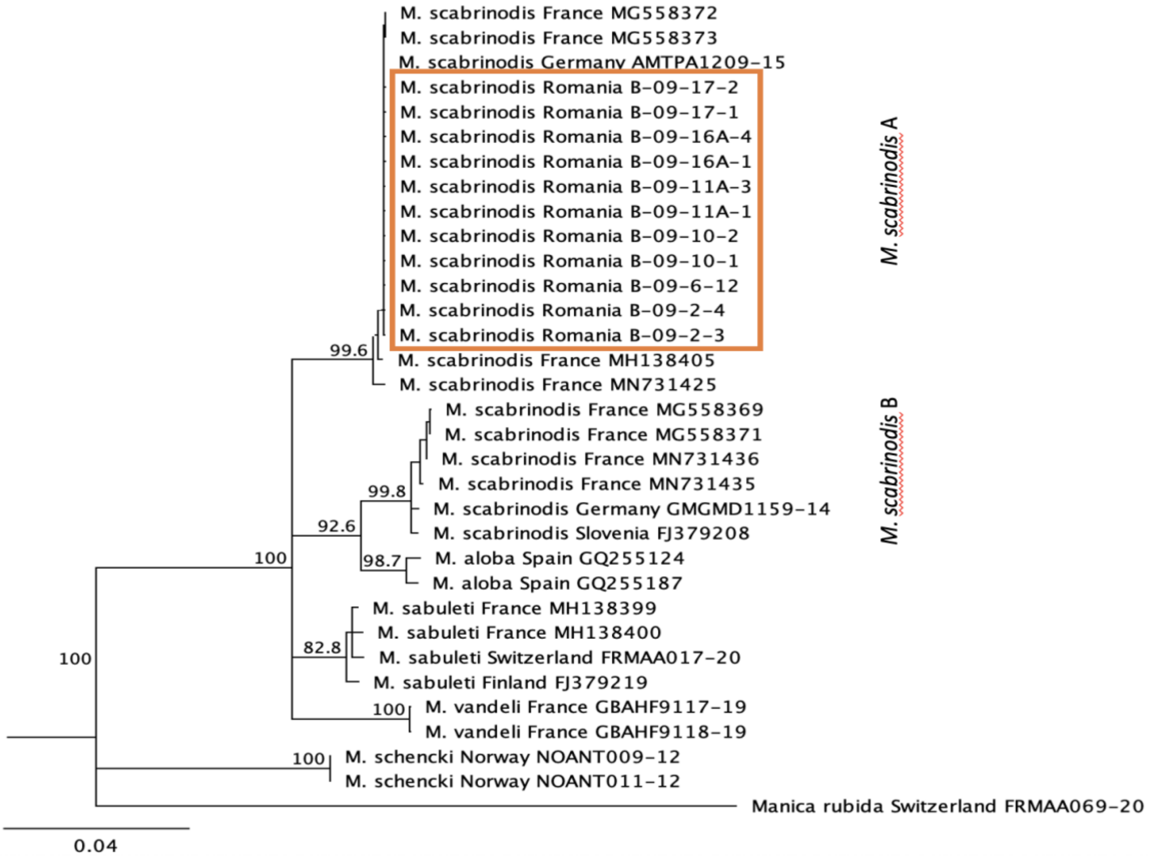

Supplementary Figure 2. Neighbor-joining consensus tree based on COI sequence data built on Tamura-Nei genetic distances. Branch lengths reflect the genetic distances and major branches have posterior probabilities assigned to them.

<u>Behavioural assays</u>

Supplementary Table 4. Summary of the outputs of statistical analyses of interaction assays reported in the main text: (a) GLMMs with colony identifier (Col ID) as random factor, (b) GLMs with treatment and Col ID as fixed factors. RD – Residual Deviation. Fig. refers to the figure where the comparison is featured. See *Statistics and reproducibility* sub-chapter in the *Materials and methods* chapter for further information.

COMPARISONS	GLMM	GLM		N	Fig
		Treatment	Col ID		
U-I corpse vs U-I extract	z = 0.6, p = 0.54*	RD = 22.38<br>p = 0.54	RD = 18.57<br>p = 0.43	18	2b,e
U-U corpse vs U-U extract	z = -0.9, p = 0.36*	RD = 15.76<br>p = 0.36	RD = 13.85<br>p = 0.59	27	2a,d
I-I corpse vs I-I extract	z = -1.96, p = 0.051*	RD = 12.10<br>p = 0.03	RD = 8.43<br>p = 0.29	27	2h,k
I-U corpse vs I-U extract	z = -0.85, p = 0.39	RD = 21.45<br>p = 0.26	RD = 7.51<br>p = 0.008	18	2g,j
I-I corpse vs U-U corpse	z = 3.7, p = 0.0002*	RD = 21.52<br>p = 0.0001	RD = 16.71<br>p = 0.3	36	2a,h
I-I corpse vs U-I corpse	z = 2.66, p = 0.007*	RD = 19.37<br>p = 0.002	RD = 14.92<br>p = 0.34	27	2b,h
U-U corpse vs I-U corpse	z = 1.83, p = 0.06*	RD = 22.58<br>p = 0.05	RD = 13.95<br>p = 0.07	27	2a,g
I-U corpse vs I-I corpse	z = 2.3, p = 0.02*	RD = 14.59<br>p = 0.01	RD = 9.65<br>p = 0.08	27	2g,h
U-U corpse vs U-I corpse	z = 1.39, p = 0.16*	RD = 27.36<br>p = 0.15	RD = 25.94<br>p = 0.49	27	2a,b
U-U extract vs I-I extract	z = 2.94, p = 0.003*	RD = 6.34<br>p = 0.01	RD = 5.58<br>p = 0.68	18	2d,k
U-I extract vs U-U extract	z = 2.49, p = 0.01*	RD = 10.79<br>p = 0.008	RD = 9.24<br>p = 0.46	18	2d,e
U-U corpse vs U-U+*n*-C23	z = 3.27, p = 0.001*	RD = 18.83<br>p = 0.001	RD = 17.93<br>p = 0.63	30	2a,c
I-U corpse vs I-U+*n*-C23	z = 1.49, p = 0.13	RD = 12.22<br>p = 0.05	RD = 5.83<br>p = 0.04	15	2g,i
U-U extract vs U-U+*n*-C23	z = -3.87, p = 0.001*	RD = 11.63<br>p = 0.001	RD = 10.61<br>p = 0.79	21	2d,f
I-U extract vs I-U+*n*-C23	z = -2.13, p = 0.03*	RD = 20.8<br>p = 0.01	RD = 11.01<br>p = 0.02	15	2j,l

* *singularity issue*

Supplementary Table 5. Statistical analysis of interaction assays: reaction of infected and uninfected workers to hexane-washed dummies supplemented with cuticular extracts. (a) GLMMs with colony identifier (Col ID) as random factor, (b) GLMs with treatment and Col ID as fixed factors. RD – Residual Deviation. See *Statistics and reproducibility* sub-chapter in the *Materials and methods* chapter for further information.

COMPARISONS	GLMM	GLM		N
		Treatment	Col ID	
I-I extract vs I-U extract	z = 1.69, p = 0.09*	RD = 18.99<br>p = 0.08	RD = 12.24<br>p = 0.03	18
I-U extract vs U-U extract	z = 1.35, p = 0.17*	RD = 14.65<br>p = 0.17	RD = 7.65<br>p = 0.07	18
I-I extract vs U-I extract	z = 0.35, p = 0.72*	RD = 15.12<br>p = 0.38	RD = 12.09<br>p = 0.72	18

* *singularity issue*

Infected workers did not differentiate between dummies with added *n*-C23 and hexane-washed dummies treated with the cuticular extracts of uninfected workers + *n*-C23 (Supplementary Table 6; Figure 2i,l). This was not the case though in uninfected live individuals (Supplementary Table 6; Figure 2c,f).

Supplementary Table 6. Statistical analysis of interaction assays: reaction of infected and uninfected workers to dummies with added *n*-C23 vs hexane-washed dummies supplemented with cuticular extracts and *n*-C23. (a) GLMMs with colony identifier (Col ID) as random factor, (b) GLMs with treatment and Col ID as fixed factors. RD – Residual Deviation. See *Statistics and reproducibility* sub-chapter in the *Materials and methods* chapter for further information.

COMPARISONS	GLMM	GLM		N
		Treatment	Col ID	
I-U + *n*-C23 *vs* I-U extract + *n*-C23	z = 0.63, p = 0.52*	RD = 11.55<br>p = 0.52	RD = 9.93<br>p = 0.44	12
U-U + *n*-C23 vs U-U extract + *n*-C23	z = 1.75, p = 0.07*	RD = 14.7<br>p = 0.03	RD = 13.7<br>p = 0.60	24

* *singularity issue*

Within colony aggression (Control experiments)

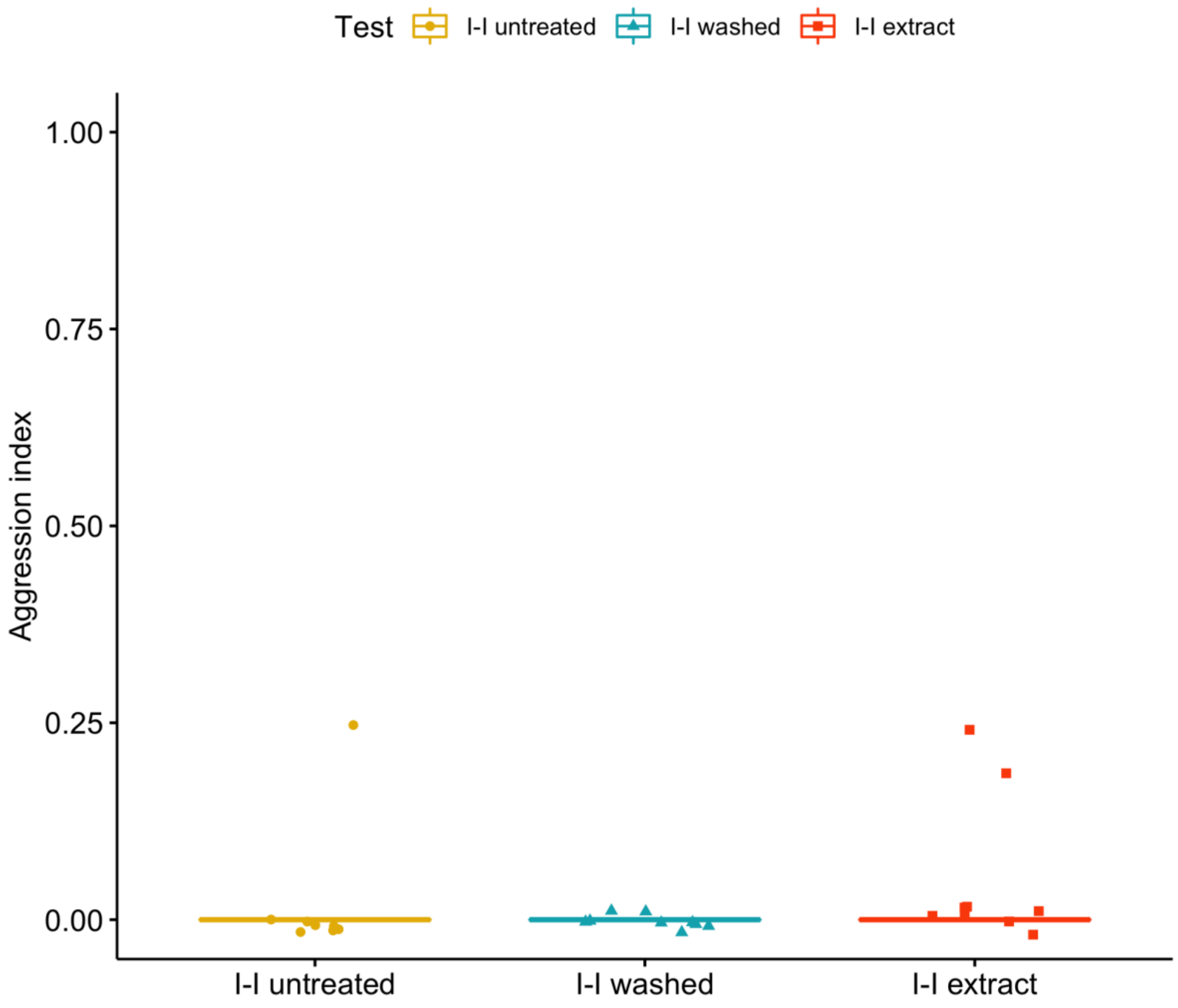

Supplementary Figure 3. Aggression indices resulting from within colony interaction assays between live infected *M. scabrinodis* workers and different types of dummies: (**a**) infected untreated corpses from the same colonies (yellow); (**b**) infected washed corpses from the same colonies (blue); (**c**) dummy + infected CHC from the same colonies (red).

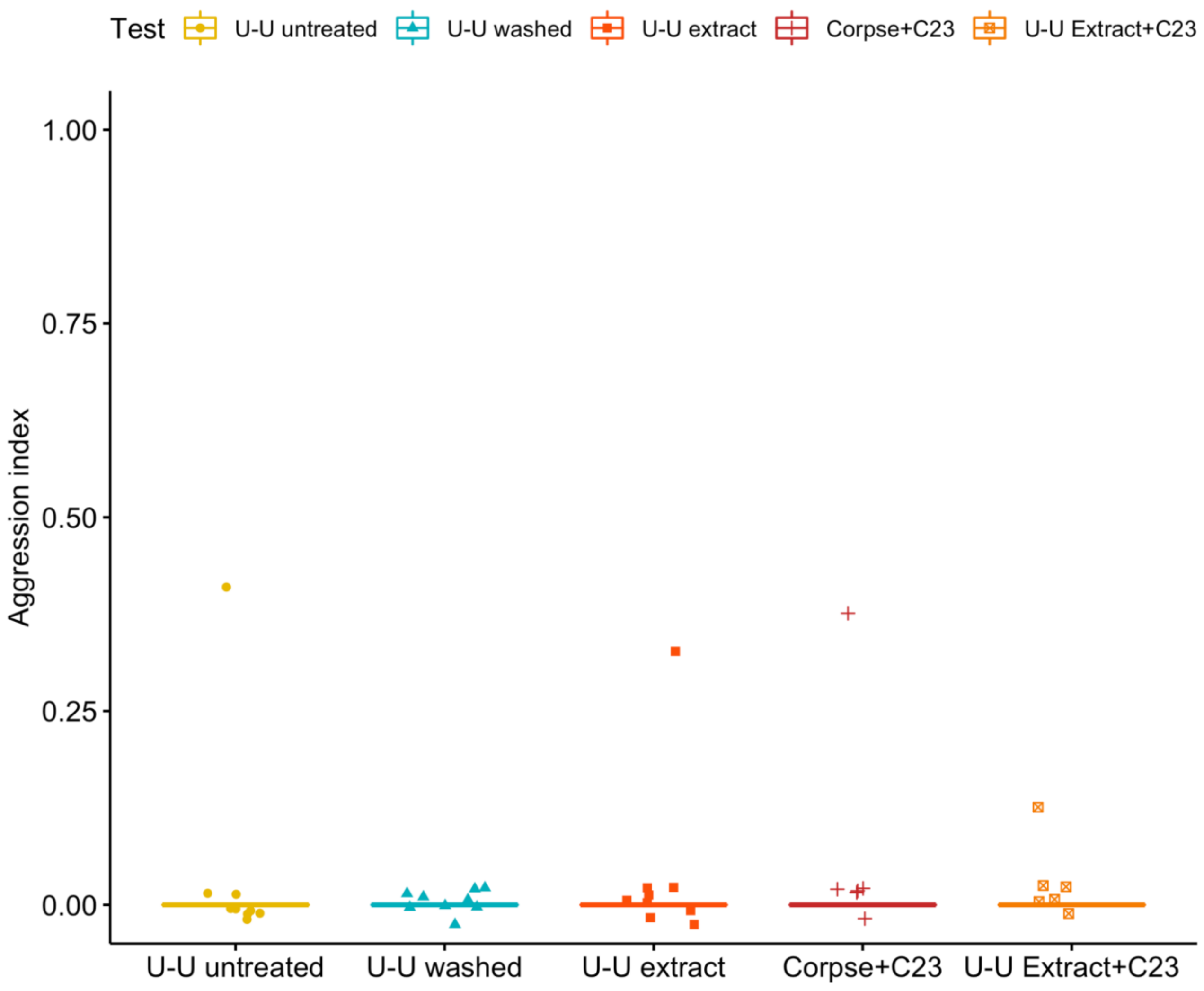

Supplementary Figure 4. Aggression indices resulting from within colony interaction assays between live uninfected *M. scabrinodis* workers and different types of dummies: (**a**) uninfected untreated corpses from the same colonies (yellow); (**b**) uninfected washed corpses from the same colonies (blue); (**c**) dummy + infected CHC from the same colonies (red); (**d**) uninfected corpse + *n*-C23 from the same colonies (dark red); (**e**) dummy + uninfected CHC + *n*-C23 (orange) from the same colonies.

References

1. Csata E, Timuș N, Witek M, Casacci LP, Lucas C, Bagnéres AG. Sztencel-Jabłonka A, Barbero F, Bonelli S, Rákosy L, Markó, B. 2017. Lock-picks: fungal infection facilitates the intrusion of strangers into ant colonies. *Sci. Rep.* **7**, 46323.
