# Supplementary material for: Non-lethal fungal infection could reduce aggression towards strangers in ants
Source: Commun Biol. 2023 Feb 16;6:183. doi: 10.1038/s42003-023-04541-7 (PMC9935638; doi:10.1038/s42003-023-04541-7)
Supplement: Supplementary file 2 — Description of Additional Supplementary Files [file 42003_2023_4541_MOESM2_ESM.pdf]

Description of Additional Supplementary Files

File name: Supplementary Code 1
Description: R scripts for figure generation and statistical analyses.
