# Supplementary material for: Non-lethal fungal infection could reduce aggression towards strangers in ants
Source: Commun Biol. 2023 Feb 16;6:183. doi: 10.1038/s42003-023-04541-7 (PMC9935638; doi:10.1038/s42003-023-04541-7)
Supplement: Supplementary file 4 — Reporting Summary [file 42003_2023_4541_MOESM4_ESM.pdf]

nature portfolio

Corresponding author(s): Enikő Csata and Luca Pietro Casacci

Last updated by author(s): 11.02.2022

Reporting Summary

Nature Portfolio wishes to improve the reproducibility of the work that we publish. This form provides structure for consistency and transparency in reporting. For further information on Nature Portfolio policies, see our Editorial Policies and the Editorial Policy Checklist.

Statistics

For all statistical analyses, confirm that the following items are present in the figure legend, table legend, main text, or Methods section.

n/a	Confirmed	
☐	☒	The exact sample size (n) for each experimental group/condition, given as a discrete number and unit of measurement
☐	☒	A statement on whether measurements were taken from distinct samples or whether the same sample was measured repeatedly
☐	☒	The statistical test(s) used AND whether they are one- or two-sided<br>*Only common tests should be described solely by name; describe more complex techniques in the Methods section.*
☐	☒	A description of all covariates tested
☐	☒	A description of any assumptions or corrections, such as tests of normality and adjustment for multiple comparisons
☐	☒	A full description of the statistical parameters including central tendency (e.g. means) or other basic estimates (e.g. regression coefficient) AND variation (e.g. standard deviation) or associated estimates of uncertainty (e.g. confidence intervals)
☐	☒	For null hypothesis testing, the test statistic (e.g. F, t, r) with confidence intervals, effect sizes, degrees of freedom and P value noted<br>*Give P values as exact values whenever suitable.*
☒	☐	For Bayesian analysis, information on the choice of priors and Markov chain Monte Carlo settings
☒	☐	For hierarchical and complex designs, identification of the appropriate level for tests and full reporting of outcomes
☒	☐	Estimates of effect sizes (e.g. Cohen's d, Pearson's r), indicating how they were calculated

Our web collection on statistics for biologists contains articles on many of the points above.

Software and code

Policy information about availability of computer code

Data collection	Samples of cuticular hydrocarbons (CHC) were analysed on a Shimadzu GC2010 gas chromatograph (GC) connected to a QP2010 plus mass spectrometer (MS; Shimadzu, Duisburg, Germany). The GC was equipped with a non-polar capillary column (BPX-5, 30m length, 0.25mm inner diameter, 0.25 µm film thickness; SGE Analytical Sciences, Milton Keynes, UK). The aggression assays were carried out using two connected transparent plastic tubes (3 cm long), which were initially separated by a small piece of red plastic foil. A single M. scabrinodis worker was placed in one tube, while the dummy was placed in the other tube. The worker was allowed to acclimatise for one minute before the red plastic foil was removed. After each assay, the plastic tubes were rinsed with ethanol (98%) and water, then wiped out and left to dry for at least 30 minutes. Assays were carried out with both infected and uninfected workers with three replicates per combination, and new individuals were used for each test.
Data analysis	All statistics were performed using R 4.0.2. Aggression indices were analysed using a generalised linear model approach (GLM, binomial error, maximum likelihood fit) with test combinations (I–I, I–U, U–I and U–U) as fixed factors for the behavioural assays. Relative abundances of n-C23 present on the cuticle of infected and uninfected old workers and experimental ants treated with n-C23 were compared using a linear model approach (LM, maximum likelihood fit). Chemical data of infected and uninfected old workers are derived from Csata et al. 2017.GLM was performed using glm functions in the lme4 package [Bates et al. 2014]. Tukey's HSD test was used to calculate post-hoc comparisons on each factor in the model using the function glht from the R package multcomp [Hothorn et al. 2008]. The graphs were produced using the ggplot2 R package [Wickham 2009].

For manuscripts utilizing custom algorithms or software that are central to the research but not yet described in published literature, software must be made available to editors and reviewers. We strongly encourage code deposition in a community repository (e.g. GitHub). See the Nature Portfolio guidelines for submitting code & software for further information.

Data

Policy information about availability of data

All manuscripts must include a data availability statement. This statement should provide the following information, where applicable:

- Accession codes, unique identifiers, or web links for publicly available datasets
- A description of any restrictions on data availability
- For clinical datasets or third party data, please ensure that the statement adheres to our policy

All data generated or analysed during this study are included in this published article (and its supplementary information files).

Field-specific reporting

Please select the one below that is the best fit for your research. If you are not sure, read the appropriate sections before making your selection.

☐ Life sciences ☐ Behavioural & social sciences ☒ Ecological, evolutionary & environmental sciences

For a reference copy of the document with all sections, see nature.com/documents/nr-reporting-summary-flat.pdf

Ecological, evolutionary & environmental sciences study design

All studies must disclose on these points even when the disclosure is negative.

Study description	To prove the role of CHC-profile in nestmate/non-nestmate discrimination in M. scabrinodis, we initially tested (1) the response of ants to hexane-washed dummies (see below for further details; N = 36). Then we performed unmanipulated assays (2) in which the reactions of live workers were tested to infected (I) and uninfected (U) dummies (N = 54) to assess the baseline aggression towards non-nestmates for both cases
Research sample	Ant species: Myrmica scabrinodis, from infected and uninfected colonies, collected all from the same population in Romania, Europe<br>Ectoparasitic fungus: Rickia wasmannii - the ant species was infected with this fungal organism
Sampling strategy	Colonies were collected from the field, behavioral observations of infected and uninfected ants were performed in laboratory with randomly chosen individuals
Data collection	Dyadic behavioural tests were performed after four days of acclimatisation under laboratory conditions with worker ants from infected and uninfected colonies (figure 1). Since in dyadic tests with live workers it is hard to tell apart the reactions of the two individuals, we used one live worker and one freshly killed non-nestmate conspecific corpse (dummy) in each assay. This allowed us to precisely characterize the response of the live worker. To eliminate or to reduce observational biases, the observer did not know the infection status of the live worker. The observer was Enikő Csata.
Timing and spatial scale	Colonies of the ant Myrmica scabrinodis, both infected (I, N = 6) and uninfected (U, N = 6), were collected from the same population near Cluj-Napoca, Romania (46.92N, 23.73E, 410–460 m a.s.l.)
Data exclusions	No data were excluded from the manuscript
Reproducibility	All attempts to repeat the experiment were successful.
Randomization	The observations were randomized, and the observer did not know the infection status of the live individual and the dummy.
Blinding	To eliminate or to reduce observational biases, the observer did not know the infection status of the live worker.

Did the study involve field work? ☒ Yes ☐ No

Field work, collection and transport

Field conditions	Myrmica scabrinodis is widely distributed throughout Europe, being found in a very wide range of habitats, but it is most commonly found in grasslands, meadows, open forests, and woodland. Their nests are usually under pieces of wood or stones or built directly into the soil. Like most Myrmica species, M. scabrinodis forms small colonies of a few hundred workers and one to many functional queens, being facultative polygynous
Location	Cluj-Napoca, Romania (46.92N, 23.73E, 410–460 m a.s.l.)
Access & import/export	We could reach the collection sites via car from Cluj-Napoca (Romania). No permit is necessary to collect the ant species Myrmica scabrinodis in Romania.
Disturbance	Ant colonies were collected from the field by careful removal of the colonies, with soil ball, without disturbing other areas of the habitat In the studied population, the species is very abundant, thus there isn't any risk for population collapse. During sampling plant

communities, or other animal species were not disturbed.

Reporting for specific materials, systems and methods

We require information from authors about some types of materials, experimental systems and methods used in many studies. Here, indicate whether each material, system or method listed is relevant to your study. If you are not sure if a list item applies to your research, read the appropriate section before selecting a response.

Materials & experimental systems

n/a	Involved in the study
☒	☐ Antibodies
☒	☐ Eukaryotic cell lines
☒	☐ Palaeontology and archaeology
☐	☒ Animals and other organisms
☒	☐ Human research participants
☒	☐ Clinical data
☒	☐ Dual use research of concern

Methods

n/a	Involved in the study
☒	☐ ChIP-seq
☒	☐ Flow cytometry
☒	☐ MRI-based neuroimaging

Animals and other organisms

Policy information about studies involving animals; ARRIVE guidelines recommended for reporting animal research

Laboratory animals	The study did not involve laboratory animals.
Wild animals	Myrmica scabrinodis ant workers were observed in laboratory conditions. After field collections, experimental colonies of M. scabrinodis were kept in plastic boxes with a food mixture of sugar and proteins provided daily. Live ants were kept for further studies, dummies were used for CHC analyses.
Field-collected samples	Experimental colonies of M. scabrinodis were kept in plastic boxes (16cm × 10cm × 5cm) with wet foam bricks under controlled lab conditions (20°C, 12 L:12D cycles).
Ethics oversight	The project, the topic and its methods were approved by the Babes-Bolyai University based on its internal regulations and policies in the frame of PN-II-RU-TE-2014-4-1930.

Note that full information on the approval of the study protocol must also be provided in the manuscript.
